# Comparative Efficacy of Wood Ash and Leaf Powder of *Aquaria salicifolia* Against *Sitophilus zeamais* and *Acanthoscelides obtectus*

**DOI:** 10.1155/sci5/8531268

**Published:** 2026-01-16

**Authors:** Bertilla Beizuh Bua, Jean Wini Goudoungou, Katamssadan Tofel Haman, Daniel Kosini, Raoul Borkeum Barry, Elias Nchiwan Nukenine

**Affiliations:** ^1^ Department of Zoology, Faculty of Science, University of Bamenda, Bamenda, Cameroon, unibda.net; ^2^ Department of Phytosanitary Protection, Institut Supérieur d’Agriculture, du Bois, de l’Eau et de l’Environnement, University of Bertoua, Bertoua, Cameroon, univ-bertoua.cm; ^3^ Department of Biological Sciences, Faculty of Science, University of Garoua, Garoua, Cameroon; ^4^ Department of Life Sciences, Higher Teacher Training College of Bertoua, University of Bertoua, Bertoua, Cameroon, univ-bertoua.cm; ^5^ Department of Biological Sciences, Faculty of Science, University of Ngaoundere, Ngaoundere, Cameroon, univ-ndere.cm

**Keywords:** *Acanthoscelides obtectus*, *Aquaria salicifolia,* leaf powder, *Sitophilus zeamais*, wood ash

## Abstract

Maize and beans are very important sources of proteins and carbohydrates, and these grains are widely cultivated and stored for further use and consumption. During their storage, maize and beans are seriously attacked by beetle pests, namely, *Sitophilus zeamais* and *Acanthoscelides obtectus,* respectively. The pest management of these beetles is mostly carried out by the use of chemical insecticides, which are not environmentally friendly. In this regard, the insecticidal efficacy of *Aquaria salicifolia* leaf powder and wood ash was assessed on *S. zeamais* and *A. obtectus* concerning their effects on adult mortality, progeny inhibition, repellence, population increase and grain damage in the fluctuating laboratory conditions. Each biosubstance was mixed with grain at different contents (5, 10, 20 and 40 g/kg). Mortality was determined for 1, 3, 7, 10 and 14 days post‐treatment. All treatments were displayed on the shelves in the darkness. Four repetitions were carried out for each test, and the experiment was done in a complete randomized block design. The substances significantly caused mortality in the study coleopteran compared to the negative control, with wood ash being more effective than the leaf powder. 40 g/kg of wood ash caused 100% mortality to the bruchids after 10 days of exposure, while 40 g/kg of leaf powder caused 100% mortality after 14 days of exposure. For the weevils, the leaf powder was less effective as the highest dosage causing 37.50% in 14 days whereas wood ash caused 73.75% mortality in the same period. The production of F_1_ progeny and rate of population increase as well as percentage of damaged grains and weight losses were reduced significantly by both substances, which were also repellent. Considering these findings, *A. salicifolia* products could favourably be used as an alternative to chemically synthesized insecticides employed in the conservation of maize and beans during storage.

## 1. Introduction

Cereals and pulses constitute the most consumed grains by humans and animals around the world. Those grains fulfil most of the dietary needs of the population across the tropics, especially in sub‐Saharan Africa [1, 2]. Among these grains, maize and beans remain the most consumed by the population in African countries, including Cameroon (Central Africa).

Maize (*Zea mays*) is the most successful crop and is extensively cultivated as a cereal grain. It is one of the most adaptable plants in different agroecological zones. Widely, maize is recognized as a very important cereal thanks to its highest optimal genotype. Maize is the only food cereal plant that can be cultivated in different seasons and agroecological zones and has various uses. It importantly serves as raw material in the industry and offers huge possibilities for enrichment [3]. Beans (*Phaseolus vulgaris*) are a vital legume crop cultivated and directly consumed by humans, and they have a considerable commercial value far superior to all other edible legumes [3–5]. The dried seeds of *P. vulgaris* contain several proteins, which are an important source of proteins in the human diet for millions of people in the tropics. In addition, these edible pulses compensate for the lack or the low content of amino acids methionine and cysteine due to the diet based on rice, maize and other cereals [4].

Beans and maize are consumed and used throughout the year, whereas their cultivation is done once per year, but the availability of these grains needs to be ensured during the whole period for their use and/or consumption. Accordingly, the storage of these grains is imperative to satisfy the demand. The storage of these grains faces several constraints. Among these, the infestation by *Sitophilus zeamais* and *Acanthoscelides obtectus* represents the most damaging factor on maize and beans, respectively [6, 7]. By their harmful action on stored products, these coleopteran pests are responsible for serious injury, inducing considerable economic losses in the storage activity.

Widely diffused over the globe, maize weevil, *S. zeamais* (Coleoptera: Curculionidae), and bean weevil*, A. obtectus* (Coleoptera: Chrysomelidae), are major pests of maize and bean grains, respectively, and other edible stored grains [8]. The weevils combined with other insects induced a loss of about of 24.5% of maize and destroyed grains by reducing nutritional value and weight, diminishing germinative capacity and lowering commercial value [9–11]. The losses caused by these beetles are really considerable; for example, in Cameroon, Nukenine et al. [12] observed that *S. zeamais* caused more than 33% damage in maize grain during 5 months of storage. This situation could be the same in the rest of the country, as the storage structures remained less developed.

*Sitophilus zeamais* commonly known as the maize weevil is among the most devastating stored grain pests and also other processed and unprocessed products during storage in Africa, especially in sub‐Saharan regions. *Sitophilus zeamais* provokes the qualitative and quantitative losses to stored products. During storage, this pest induces considerable loss of grain weight without appropriate protection methods, ranging within 20%–90% for stored maize [12], and the intensity of damage depends on several factors, including storage facilities, physical and chemical characteristics of the stored products. *Acanthoscelides obtectus* commonly known as bean bruchid is the main insect pest of stored beans. This bruchid species feeds on vetches, beans and other leguminous plants [13, 14].

In order to ensure proper conservation of these useful agricultural products against insect pests during both pre‐ and postharvest stages, people globally use important quantity of synthetic insecticides, which are mostly less biodegradable. However, although effective, these chemical substances are at the base of many environmental problems, such as pollution, human health risks, harmful effects on beneficial species, the occurrence and spread of resistant strains of pests, among others [15]. Furthermore, the farmers in Africa mostly have low income and lack the financial means to purchase the appropriate pesticides. In addition, they do not have enough skills for handling and managing the pesticides properly. These disadvantages of synthetic insecticides have motivated the serious look for alternative methods and substances, which are less harmful to human health and less detrimental and even protective to the environment [16]. Indeed, plant products have been identified as candidate substances capable of fulfilling this need. In this issue, many studies are focused on determining these plants endowed with pesticide properties, identifying and isolating the compounds in the plants responsible for the insecticidal activity [17].

*Aquaria salicifolia* belongs to the family Ericaceae. This family is a large cosmopolitan plant family, and it is represented by 124 genera and 4100 species [18]. The plants of these taxa are importantly distributed in the temperate zones of the world and also in the tropical mountains of Southeast Asia and the American continent [19]. The Ericaceae plants are characterized by the production of many edible berries that can be cultivated. However, some species of this family are very toxic poisons and can be used as decorative plants [19]. *Aquaria salicifolia* is found in Central Africa, Southern Africa and the flora of Madagascar. In the north‐west region of Cameroon, this plant species is particularly observed in KilumIjim (Oku) and it is also reported in the western region, especially in the Bamboutos Mountain [20, 21]. Several surveys have reported its use in traditional pharmacopoeias for curing of many diseases like diarrhoea, inflammation and as a urinary astringent and antiseptic substance [22, 23]. The surveys also pointed out the use of the leaves, roots and barks of this plant in the treatment of sexually transmitted diseases, scabies and rheumatism [22, 23]. Therefore, the present investigation sought to determine the effectiveness of *A. salicifolia* to control infestation by the maize and bean beetles, *S. zeamais* and *A. obtectus*, respectively.

## 2. Materials and Methods

### 2.1. Insect Rearing

*Sitophilus zeamais* and *Acanthoscelides obtectus* adults were collected from a colony reared in the Biological Sciences Laboratory of the Faculty of Science of Bamenda University, Cameroon. One hundred maize weevils were introduced into 250‐g maize grains in each of 5 glass jars with perforated lids. Maize weevils were reared in mass on whole, clean, unbroken and disinfected maize in 900‐mL glass jars. The culture was maintained in the darkness under fluctuating laboratory conditions (temperature (T) = 23.35°C ± 2.07°C; relative humidity (RH) = 75.12% ± 6.50%) and used as a source of *S. zeamais* for the different bioassays. Temperature and humidity during the study were recorded by the data logger (Data logger Model EL‐USB‐2, LASCAR, China). The bruchids were cultured on cleaned and unbroken common bean (*Phaseolus vulgaris*) in the same type of jars used for maize weevil’s culture and kept under the same laboratory conditions. Since the lifespan of *A. obtectu*s adult is short, to allow a better assessment of the effectiveness of the plant materials, all adult insects used were ≤ 2 days old.

### 2.2. Preparation of Leaf Powder and Wood Ash

The plant identification was done at the National Herbarium in Yaounde, Cameroon, with voucher number N°33530SRF Cam. *Aquaria salicifolia* green leaves were collected in February 2022 in the south‐west region of Cameroon, precisely at Lebialem. The leaves were shade‐dried at room temperature until they became crisp. Then, those leaves were ground into powder using a locally made pestle and mortar. The stem and branches of the same plant were collected. Woods were air‐dried until the moisture was completely lost and then burnt. The obtained ash was sieved using a sifter of 0.3‐mm mesh size and packaged in a glass jar. Leaf powder and wood ash were conserved in the labelled and nontransparent glass jar and kept in a freezer (at −4°C) until their use in the different bioassays.

### 2.3. Grains Used for Bioassays

The white grain of locally cultivated maize was used. Before experimentation, damaged grains, pieces of stone, sand and other foreign materials were removed manually from the stock. The maize grains were conserved in the freezer at −20°C for 14 days for better disinfestation. After the grains had been disinfested from all types of possible living organisms, the grains were conserved in ambient laboratory conditions for 14 days to allow its acclimatization. After all these steps, the grains of maize were ready to be used as substrate for insect cultures and tests. The variety of common beans (*Phaseolus vulgaris*) used during the experimentation was the *Medino* variety, obtained from a smallholder stock in the north‐west. The same procedure used for maize grains was applied to beans. The moisture contents were 11.81% and 12.32% for bean and maize, respectively.

### 2.4. Mortality Bioassay

Different quantities (0.25, 0.5, 1 and 2 g) of leaf powder and wood ash of *A. salicifolia* were separately added to 50 g of common beans/maize contained in glass jars and covered with perforated lids to allow aeration. These quantities of 0.25, 0.5, 1 and 2 g of products in 50g of grain corresponded to 5, 10, 20 and 40 g/kg in terms of concentration. The mixture of maize or bean and plant product was manually shaken for 5 minutes to allow uniform coating of plant powder on the grains. Twenty adults of *A. obtectus* or *S. zeamais* of nondetermined sex were added to each glass jar. The negative control was made up by infesting the beans and maize with 20 insects each but without plant material. For positive control, 0.5 g/kg of Gredox (Pirimiphos‐methyl, organophosphate) was added to 50g of common beans/maize and shaken manually to allow uniform coating of the chemical on grains. 20 adults of *A. obtectus*/*S. zeamais* were added to the mixture. After adding insects, the jars were covered and displayed on shelves in the same ambient laboratory conditions (*T* = 23.35 ± 2.07°C; RH = 75.12 ± 6.50%). Each test was subject to four replications, and the experiment was displayed in a complete randomized block design. In order to assess mortality, the observations were carried out 1, 3, 7, 10 and 14 days postinfestation. During these observations, the dead and live *A. obtectus* and *S. zeamais* were counted and recorded. Insects not moving even when they were touched several times with the entomological forceps were considered dead.

### 2.5. Progeny Production Test

After evaluation of 14 days of mortality, the insects were removed from the jars. The grains were kept in the jars displayed on the shelves. Each jar containing the grains previously kept was observed for F_1_ emergence. The observations were carried out once every week. The F_1_ adult insects were counted starting from the first progeny emergence. The counting was cancelled when F_1_ progeny emergence decreased and almost completely stopped in order to avoid overlapping with the next emergence. After each counting session, the insects were removed from the jars and recorded. The inhibition rate of F_1_ progeny (%IR) was calculated as follows:

(1) %IR=Cn−TnCn×100,

where Cn is the number of emerged insects in the untreated jar and Tn is the number of insects in the treated jar.

### 2.6. Repellency Test

The area preference method according to McDonald et al. [24] was employed to determine the repellent action of the leaf powder of *A. salicifolia* on *A. obtectus* and *S. zeamais*. Test arenas consisted of a 9‐cm Petri dish. The Petri dish was divided into two using a filter paper. Different concentrations (5, 10, 20 and 40 g/kg) of powder were used. Maize/bean treated with leaf powder was put on one side of the Petri dish as uniformly as possible. The other side was made by nontreated maize/bean. About 20 nondetermined adult insects of each species were released separately at the centre of the filter paper. The Petri dishes were covered and left under the same ambient laboratory conditions as previous tests. The experiment was submitted to four replications. The number of insects present in the control (*N*_*C*_) and treated (*N*_*T*_) grains was counted and recorded after 24 h of exposure. Percentages of repellence (PRs) were calculated as follows [24]:

(2) PR=NC−NTNC+NT×100.

### 2.7. Population Increase and Damage Bioassay

Different quantities (1, 2 and 4 g) of leaf powder/wood ash of *A. salicifolia* were added to 100 g of common bean/maize grains separately, corresponding to the concentrations of 10, 20 and 40 g/kg. The jars with bean/maize and plant powder/ash were hand shaken to permit uniform coating of powder on the grain surface. Then, 20 adults of *A. obtectus* and 20 adults of *S. zeamais* of undetermined sex were added to the glass jars containing bean and maize, respectively. The control was made by the same quantity of grain and the same number of insects but without plant powder/wood ash. The experiments were subject to four repetitions, displayed on the shelves and stored for 3 months. The experiment was carried out in a complete randomized block design and in the same fluctuating conditions. After this period, the number of insects for each dosage was counted and recorded in order to determine the population increase. At the same time, damage was determined by measuring and counting the number of damaged and undamaged grains according to Adams and Schulten [25]:

(3) Weight loss%=Wu×Nd−Wd×NuWuNd+Nu×100,

where Wu is the weight of undamaged grain, Nd is the number of damaged grains, Wd is the weight of damaged grain, and Nu is the number of undamaged grains.

### 2.8. Data Analysis

The correction for control mortality was carried out using Abbott’s formula [26] before analysis of variance (ANOVA) and probit analysis. Different transformations of data have been carried out before the ANOVA procedure. Data were log transformed (*x* + 1) for cumulative corrected mortality, reduction in F_1_ progeny, damage and percentage weight loss, whereas data on the number of F_1_ progeny were arcsine‐transformed (square root (*x*/100)) [27]. The lethal dosages (LDs) of *S. zeamais* and *A. obtectus* at 1, 3, 7, 10 and 14 days after treatment were computed using the probit analysis [28]. The same procedure was also used to calculate the effective dosage 50 (ED_50_) and 95 (ED_95_); these dosages represent the dosages that induce 50% and 95% inhibition of F_1_ progeny production. Tukey’s test (*p* = 0.05) was employed for means separation.

## 3. Results

### 3.1. Toxicity

#### 3.1.1. Adult Mortality

**Figure 1:**
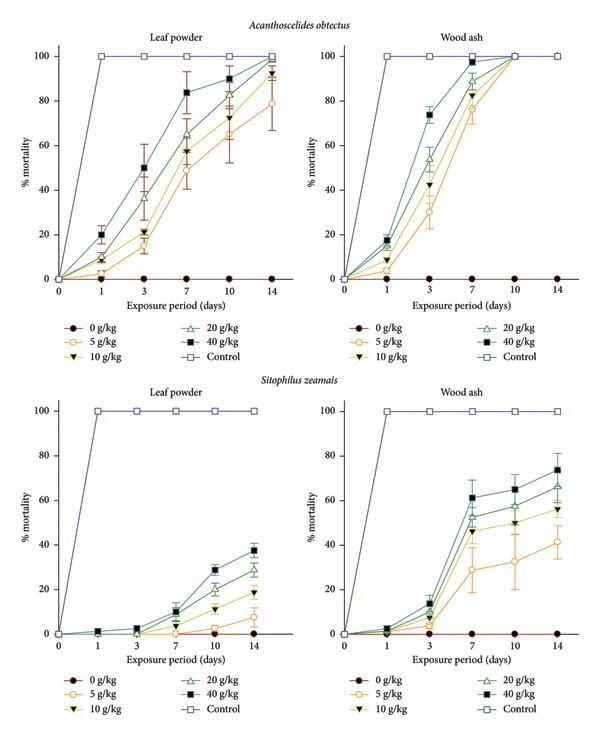
Cumulative mortality induced by leaf powder and wood ash of *Aquaria salicifolia* on *Acanthoscelides obtectus* and *Sitophilus zeamais* adults within 1, 3, 7, 10 and 14 days of exposure in the ambient laboratory conditions (*T* = 23.35°C ± 2.07°C; RH = 75.12% ± 6.50%).

The mortality of *S. zeamais* and *A. obtectus* was affected differently by the different dosages of *A. salicifolia* products and periods of exposure (Figure 1)*.* Leaf powder and wood ash of *A. salicifolia* significantly induced mortality of *A. obtectus* adults. This mortality increased with dosage and exposure time for each product. The efficacy was not the same for the two tested products. The highest mortality rate was reached by the highest dosage (40 g/kg) of *A. salicifolia* wood ash on *A. obtectus* adults, which was 100% mortality after 10 days of exposure, while the leaf powder induced 100% mortality after 14 days of exposure at 40 g/kg. The highest dose (40 g/kg) recorded 20.00% mortality after one day for leaf powder, while wood ash recorded 17.50% mortality after one day of exposure. Significant mortality of *S. zeamais* adults was also observed with both products of *A. salicifolia* (Figure 1). A low mortality rate was recorded with *A. salicifolia* leaf powder on *S. zeamais* compared to that induced on *A. obtectus*. After one day of exposure, the highest content (40 g/kg) recorded 2.50% mortality of *S. zeamais* with wood ash, while leaf powder recorded 1.25%. The highest mortality rate of *S. zeamais* (73.75%) was achieved by the highest content (40 g/kg) after 14 days of exposure to wood ash, while for leaf powder, the highest dosage recorded 37.50%.

#### 3.1.2. Relative Dose Response

The LD_50_ and 95 (LD_95_) decreased when the exposure periods increased (Table 1). For *A. obtectus*, the LC_50_ of wood ash and leaf powder was 14.12 and 40.68 g/kg, respectively, in 3 days. However, in 10 days, the LD_50_ of the two powders in the same order was 1.40 and 6.18 g/kg, respectively. The comparable performance was observed with LD_95._ The lowest values for both LD were attained at 14 days of exposure, and lethal content for wood ash could not be calculated due to the complete mortality (100%) at all the dosages, while leaf powder recorded 2.39 g/kg (Table 1). For *S. zeamais*, the LD_50_ of wood ash and leaf powder was 129.558 and 185.815 g/kg, respectively, in 3 days. The different LD values decreased when the exposure period increased for the two plant powders, as previously observed with *A. obtectus*. The lowest values on *S. zeamais* were recorded within 14 days of exposure, where LD_50_ was 7.71 and 67.23 g/kg for wood ash and leaf powder, respectively.

**Table 1:** Toxicity parameters of leaf powder and wood ash of *Aquaria salicifolia* on *Sitophilus zeamais* and *Acanthoscelides obtectus* under the fluctuating laboratory conditions (*T* = 23.35 ± 2.07°C; RH = 75.12% ± 6.50%).

Products	*R* ^2^	Slope ± SE	LD_50_ (95% FL) (g/kg)	LD_95_ (95% FL) (g/kg)	*χ* ^2^
*Sitophilus zeamais*					
3d					
Wood ash	0.31	0.71 ± 0.14	129558 (181.18; 325.108)	—	50.60^∗∗∗^
Leaf powder	0.36	3.018 ± 1.227	185.815 (89.169; 1107.677)	651.825 (180.41; 53533240)	12.47^ns^
7d					
Wood ash	0.37	0.89 ± 0.10	17.14 (9.56; 41.26)	1214.82 (194.81; 120.10^5^)	118.74^∗∗∗^
Leaf powder	0.31	1.22 ± 0.15	361.65 (100.73; 5560797.72)	8091.24 (611.82; 5.83–10^11^)	75.27^∗∗∗^
10d					
Wood ash	0.30	0.90 ± 0.10	13.02 (4.49; 29.76)	889.27 (138.82; 122.10^7^)	159.45^∗∗∗^
Leaf powder	0.79	1.37 ± 0.13	92.76 (61.33; 192.53)	1480.98 (530.95; 9828.53)	30.73^∗^
14d					
Wood ash	0.43	0.95 ± 0.10	7.71 (2.73; 12.12)	423.59 (113.78; 104.10^3^)	98.73^∗∗∗^
Leaf powder	0.69	1.17 ± 0.11	67.23 (42.49; 173.42)	1708.29 (459.69; 32504.74)	50.76^∗∗∗^

*Acanthoscelides obtectus*					
3d					
Wood ash	0.65	125 ± 0.10	14.12 (9.74; 20.47)	295.96 (112.06; 3368.48)	94.58^∗∗∗^
Leaf powder	0.51	1.19 ± 0.10	40.68 (25.26; 141.27)	973.28 (222.08; 124449.04)	117.88^∗∗∗^
7d					
Wood ash	0.45	1.17 ± 0.13	1.40 (0.05; 3.31)	35.53 (19.87; 275.75)	93.97^∗∗∗^
Leaf powder	0.47	1.04 ± 0.10	6.18 (0.95; 10.592)	234.25 (67.73; 134987.02)	156.09^∗∗∗^
10d					
Wood ash	—	—	—	—	215.97^∗∗∗^
Leaf powder	0.25	0.99 ± 0.11	2.21 (0.00; 585)	101.75 (32.31; 2.38.10^6^)	
14d					
Wood ash	—	—	—	—	136.47^∗∗∗^
Leaf powder	0.24	2.41 ± 0.24	2.39 (0.24; 4.03)	11.46 (8.11; 32.64)	

*Note:* —: LD value could not be calculated or was too large due to inadequate mortality.

Abbreviation: LD, lethal dosage.

^ns^ *p* > 0.05.

^∗^ *p* < 0.05.

^∗∗∗^ *p* < 0.0001.

### 3.2. Inhibition of F_1_ Progeny Production

Wood ash and leaf powder of *A. salicifolia* significantly reduced F_1_ progeny production of *A. obtectus* and *S. zeamais* (Table 2). The reduction of F_1_ progeny production was dose‐dependent with the highest progeny recorded by nontreated beans (about 28insects) and maize grains (about 64 insects). The insect emergence was totally suppressed by the reference insecticide, Gredox, at its recommended dosage of 0.5 g/kg, followed by (40 g/kg). The same performance was evoked by leaf powder from the dosage of 20 g/kg and wood ash at the highest dosage (40 g/kg) on *A. obtectus*. While on *S. zeamais*, even though the inhibition was not complete, leaf powder and wood ash significantly inhibited the production of *S. zeamais* progeny by 96.59% and 76.54%, respectively. The lowest dosage (5 g/kg) of the two powders significantly induced inhibition of the F_1_ progeny for both beetles in the treated grains. They recorded about 19 (32.63% inhibition) and 9 (66.47% inhibition) of *A. obtectus* adults with leaf powder and wood ash, respectively. In maize treated with leaf powder and wood ash, they recorded 51 (26.93% inhibition) and 10 (84.10% inhibitions) of adults *S. zeamais,* respectively. The inhibition of progeny production of *A. obtectus* was higher in beans treated with leaf powder (ED_95_ = 11.67 g/kg) than wood ash (ED_95_ = 23.19 g/kg), while the reduction of progeny production of *S. zeamais* was more effective with wood ash (ED_95_ = 29.81 g/kg) than leaf powder (ED_95_ = 62.72 g/kg).

**Table 2:** Reduction of F_1_ progeny of *Acanthoscelides obtectus* and *Sitophilus zeamais* relative to the control in bean treated with leaf powder and wood ash of *Aquaria salicifolia* under the fluctuating laboratory conditions (*T* = 23.35°C ± 2.07°C; RH = 75.12% ± 6.50%).

Dosage (g/kg)	F_1_ progeny	Inhibition of F_1_ relative to the control (%)
*A. obtectus*		
Leaf powder		
0	27.25 ± 3.04^a^	0.00 ± 0.00^c^
5	18.25 ± 2.63^b^	32.63 ± 6.08^b^
1	3.25 ± 0.63^c^	86.88 ± 4.11^a^
20	0.00 ± 0.00^c^	100.00 ± 0.00^a^
40	0.00 ± 0.00^c^	100.00 ± 0.00^a^
F_(4;15)_	46.22^∗∗∗^	190.40^∗∗∗^
ED_50_ (95% FL) g/kg	6.43 (5.80; 7.00)	
ED_95_ (95% FL) g/kg	11.67 (10.66; 13.63)	
Wood ash		
0	27.25 ± 3.04^a^	0.00 ± 0.00^d^
5	8.75 ± 0.25^b^	66.47 ± 4.42^c^
10	6.25 ± 1.25^bc^	76.63 ± 4.88^bc^
20	2.50 ± 1.56^bc^	91.52 ± 4.54^ab^
40	0.00 ± 0.00^c^	100.00 ± 0.00^a^
F_(4;15)_	43.70^∗∗∗^	122.60^∗∗∗^
ED_50_ (95% FL) g/kg	—	
ED_95_ (95% FL) g/kg	23.19 (18.78; 32.83)	

*S. zeamais*		
Leaf powder		
0	63.50 ± 6.84^a^	0.00 ± 0.00^c^
5	50.75 ± 9.38^ab^	26.93 ± 6.96^bc^
10	45.00 ± 10.52^ab^	32.18 ± 9.47^bc^
20	39.75 ± 13.82^ab^	42.67 ± 17.08^ab^
40	15.25 ± 3.82^b^	76.54 ± 5.96^a^
F_(4;15)_	3.51^∗^	8.57^∗∗^
ED_50_ (95% FL) g/kg	22.99 (15.45; 34.51)	
ED_95_ (95% FL) g/kg	62.72 (45.80; 120.40)	
Wood ash		
0	63.50 ± 6.84^a^	0.00 ± 0.00^c^
5	9.75 ± 2.87^b^	84.10 ± 4.50^b^
10	7.00 ± 2.27^b^	88.75 ± 3.08^ab^
20	4.25 ± 1.03^b^	93.09 ± 1.69^ab^
40	2.25 ± 0.63^b^	96.59 ± 0.71^a^
F_(4,15)_	54.67^∗∗∗^	251.66^∗∗∗^
ED_50_ (95% FL) g/kg	—	
ED_95_ (95% FL) g/kg	29.81 (21.82; 54.42)	

*Note:* Means ± S.E. in the same column followed by the same letter do not differ significantly at *p* < 0.05 (Tukey’s test).

Abbreviation: ED, effective dosage.

^∗^ *p* < 0.05.

^∗∗^ *p* < 0.001.

^∗∗∗^ *p* < 0.0001.

### 3.3. Repellency

Leaf powder and wood ash of *A. salicifolia* significantly repelled *S. zeamais* and *A. obtectus* adults (Table 3). For *S. zeamais*, the repellence percentage decreased as the concentration increased. The lowest repellence rate (41.25%) was observed with the highest dosage (40 g/kg) of *A. salicifolia* wood ash within 24‐h period of exposure, whereas the highest PR (72.50%) was recorded with the lowest dosage of wood ash (5 g/kg). For the leaf powder, the highest dosage (40 g/kg) had the highest repellence rate (61.25%) and the lowest repellence rate was recorded with 10 g/kg (48.75%), whereas the lowest dosage (5 g/kg) recorded 56.25% (Table 3). For *A. obtectus*, the repellence rate was high but statistically the same for the two powders (*t* = −1.177 − 0.388, *p* > 0.05). Even though no statistical difference in the rate of repellence was observed among the different dosages of wood ash; the highest dosage (40 g/kg) recorded the lowest repellence rate (50.00%), while 10 and 20 g/kg recorded the highest repellence rate (61.25%). The lowest dosage (5 g/kg) of ash considerably repelled *A. obtectus* (56.25%). For the leaf powder, repellence rate was dose‐dependent with the highest dosage (40 g/kg) recording the highest repellence rate (65.00%) and the lowest dosage (5 g/kg) recording the lowest repellence rate (52.50%) (Table 3).

**Table 3:** Repellence induced by leaf powder and wood ash of *Aquaria salicifolia* on the adult of *Acanthoscelides obtectus* and *Sitophilus zeamais*, under ambient laboratory conditions (*T* = 23.35°C ± 2.07°C; RH = 75.12% ± 6.50%).

Dosage (g/kg)	Wood ash	Leaf powder	*t* value
*A. obtectus*			
5	56.25 ± 6.57^a^	52.50 ± 9.46^a^	0.301^ns^
10	61.25 ± 8.75^a^	57.50 ± 7.77^a^	0.388^ns^
20	61.25 ± 3.75^a^	60.00 ± 6.12^a^	0.174^ns^
40	50.00 ± 11.37^a^	65.00 ± 4.56^a^	−1.177^ns^
F_(3;12)_	0.43^ns^	0.52^ns^	

*S. zeamais*			
5	72.50 ± 6.29^a^	56.25 ± 7.46^a^	5.166^∗^
10	53.75 ± 3.14^ab^	48.75 ± 5.15^a^	1.414^ns^
20	52.5 ± 5.95^ab^	56.25 ± 5.54^a^	−0.429^ns^
40	41.25 ± 10.28^b^	61.25 ± 4.26^a^	−1.611^ns^
F_(3;12)_	3.52^∗^	0.81^ns^	

*Note:* Mean ± S.E. followed by the same letter in a column do not differ significantly at *p* < 0.05 (Tukey’s test).

^ns^ *p* > 0.05.

^∗^ *p* < 0.05.

### 3.4. Population Growth and Grain Damage

#### 3.4.1. Suppression of Insect Population

**Figure 2:**
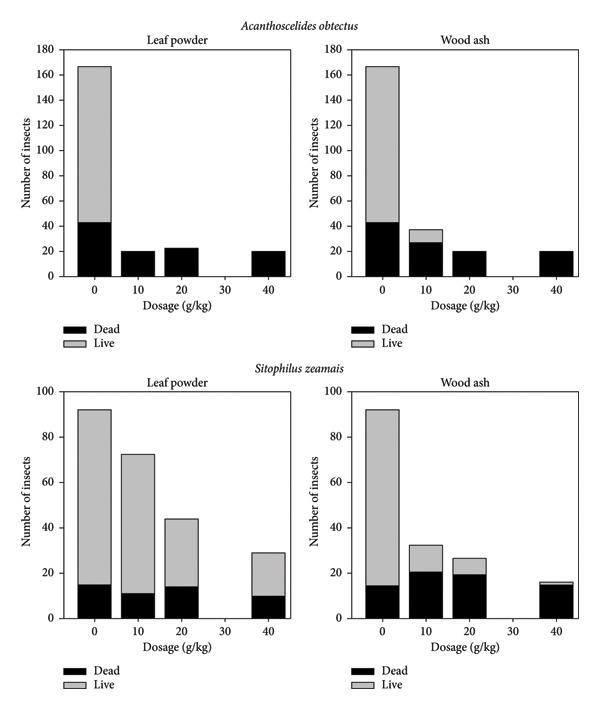
Suppression of population growth of *Acanthoscelides obtectus* and *Sitophilus zeamais* by leaf powder and wood ash of *Aquaria salicifolia* during 3 months of storage in the ambient laboratory conditions (*T* = 23.35°C ± 2.07°C; RH = 75.12% ± 6.50%).

*Aquaria salicifolia* leaf powder and wood ash significantly suppressed *S. zeamais* and *A. obtectus* population increase and reduced grain damage (Figure 2). The action of the powders was dose‐dependent, so the effectiveness improved as the dosage increased. In nontreated beans (negative control), more than 160 *A. obtectus* adults were recorded. From the dosage of 20 g/kg, the 2 *A. salicifolia* powders suppressed the *A. obtectus* population completely during the 3 months of storage; all the insects counted were found dead. The lowest content (10 g/kg) of wood ash recorded about 10 live insects (Figure 2). As observed with the bean, the nontreated maize contained the highest number of insects (about 90 adult insects). *Sitophilus zeamais* population was almost completely suppressed by the wood ash at its highest dosage (40 g/kg). The lowest dosage (10 g/kg) of both powders recorded beetle emergence compared to the high dosages. More than 25 dead beetles were recorded at the dosages of 10 and 20 g/kg of wood ash against less than 10 live ones. However, the leaf powder did not suppress the beetle population at its highest dosage (40 g/kg) completely.

### 3.5. Reduction of Grain Damage and Weight Loss

**Figure 3:**
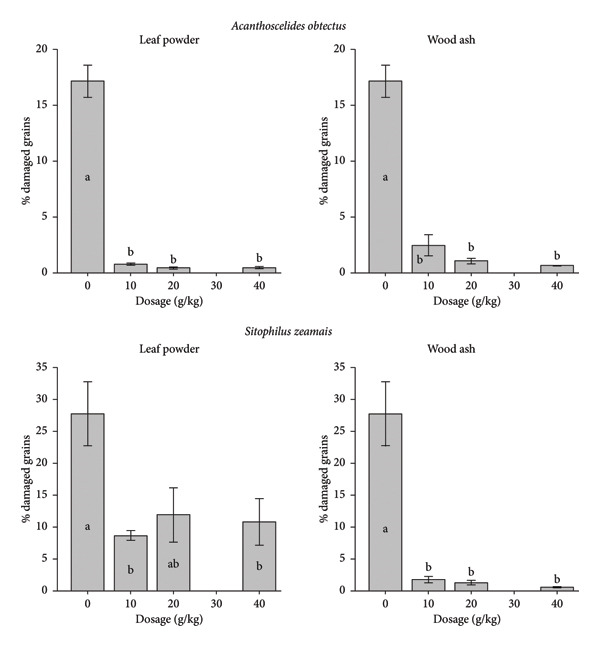
Reduction of grain damage by leaf powder and wood ash of *Aquaria salicifolia* during 3 months of storage under the attack of *Acanthoscelides obtectus* and *Sitophilus zeamais* in the ambient laboratory conditions (*T* = 23.35°C ± 2.07°C; RH = 75.12% ± 6.50%).

The damage and weight loss of bean and maize were minimized by the application of the wood ash and leaf powder of *A. salicifolia* during the 3 months of storage (Figure 3; Table 4). For perforation, the nontreated bean (0 g/kg) had 17.15% and 6.66% weight loss. The beans treated with the lowest dosage (10 g/kg) of leaf powder recorded 0.77% perforated beans with 0.20% weight loss, while the beans treated with wood ash recorded 2.46% perforated grains and 0.64% weight loss. At the highest dosage (40 g/kg) of leaf powder and wood ash, perforation rates of 0.45% and 0.66%, respectively, were observed (Figure 3), along with weight losses of 0.05% and 0.12%, respectively (Table 4). After 3 months of maize storage, the negative control recorded more grain perforation (27%) and weight loss (3.37%). From the dosages of 10 g/kg, the maize weight loss was almost totally suppressed by wood ash (< 1%), with less than 2% of grain perforated. The weight loss of maize grain was also considerably reduced at the different dosages of leaf powder; from the dosage of 10 g/kg, about 10% of grain perforation was recorded (Figure 3) with less than 2% of weight loss (Table 4).

**Table 4:** Weight loss of bean and maize treated with the leaf powder and wood ash of *Aquaria salicifolia* and stored for 3 months in the ambient laboratory conditions (*T* = 23.35°C ± 2.07°C; RH = 75.12% ± 6.50%).

Dosage (g/kg)	Leaf powder	Wood ash
*Acanthoscelides obtectus*		
0	6.66 ± 0.57^a^	6.66 ± 0.57^a^
10	0.20 ± 0.005^b^	0.64 ± 0.16^b^
20	0.11 ± 0.02^b^	0.15 ± 0.06^b^
40	0.05 ± 0.02^b^	0.12 ± 0.03^b^
F_(3;12)_	128.85^∗∗∗^	112.89^∗∗∗^

*Sitophilus zeamais*		
0	3.37 ± 0.47^a^	3.37 ± 0.47^a^
10	1.62 ± 0.47^a^	0.66 ± 0.25^b^
20	1.92 ± 0.79^a^	0.48 ± 0.11^b^
40	1.89 ± 0.34^a^	0.24 ± 0.02^b^
F_(3;12)_	2.06^ns^	28.94^∗∗∗^

*Note:* Mean ± S.E. followed by the same letter in a column do not differ significantly at *p* < 0.05 (Tukey’s test).

^ns^ *p* > 0.05.

^∗∗∗^ *p* < 0.0001.

## 4. Discussion

The leaf powder and wood ash of *A. salicifolia* showed high insecticidal properties against *S. zeamais* and *A. obtectus*. Significant mortality in *A. obtectus* and *S. zeamais* has been induced by the leaf powder and wood ash. This mortality varied according to the types of plant products. The leaf powder showed less effectiveness, whereas wood ash induced significant mortality. The effectiveness of these plants depended on dose, and the mortality increased with increasing period of exposure and quantity of plant products. As the exposure period prolonged, the contact with insecticidal product increased; consequently, higher dosages led to greater quantities of active ingredients. The insecticidal potential of these plant powders could be assigned to the phytochemical compounds contained in these products. The increase in mortality imputed to the period of exposure and dosage could be attributed to the quantity of active compounds picked up by the insects. Many plant extracts have showed their capacity to induce mortality against *S. zeamais* and *A. obtectus*. Powders of *Plectranthus glandulosus*, *Chenopodium ambrosioides* and *Tephrosia vogelii* leaf powder caused significant mortality of *S. zeamais* and *A. obtectus* adults [29, 30]. The insecticidal efficacy of plant products could be allocated to their repellence, digestive poisoning and respiratory system disturbance through the obtrusion of spiracles [31]. The mode of action of plant powders that induce insect pests’ mortality includes the abrasion of the cuticle that induces desiccation and the obstruction of the spiracle by the dust particles [32, 33]. Generally, in the present study, wood ash was more effective than the leaf powder. This difference may be linked to differences in their chemical compounds. The screening revealed that wood ash contained more calcium and phosphorus than the leaf powder that increases the abrasive action of ash. As observed by Ajiboye et al. [33], the bioactive compounds of botanical powders are limited by other inherent inert materials that could explain the low efficacy of leaf powder compared to ash.

For a good conservation of stored grains, a protectant whether plant products or any natural substance must have the ability to suppress pest population and at the same occasion reducing loss. The leaf powder and wood ash of *A. salicifolia* suppressed the population of *S. zeamais* and *A. obtectus*, and lower grain damage, as well as weight losses of maize and bean. The powders used in the present work controlled insect population by several modes of action, such as antifeedant property, moulting inhibition, growth inhibition, fecundity loss and respiratory disturbance [34, 35]. The present findings are in line with several studies including Goudoungou et al. [29], Mukanga et al. [36], Adedire et al. [37], and Ileke and Oni [38], where botanicals were effective for the control of different insect pest species in stored grains. The compounds in the plant products may exert antifeedant, toxic and regulatory activity, which act on the insect physiological processes, and possess the capacity to reduce the contact with grain and the injuries caused by phytophagous insects. The plant products such as leaf powder of *Plectranthus glandulosus* and wood ash of *Hymenocardia acida* showed their efficacy to reduce damage caused by *S. zeamais* on maize grain by greatly suppressing mass loss and grain damage in comparison with the nontreated one at the dosage of 40 g/kg [29]. The reduction of grain damage might be linked to the suppression of *S. zeamais* and *A. obtectus* population growth. This suppression of pest population could be imputed to ovicidal, repellent, larval mortality or even the disturbance of postembryonic development [39], in addition to the feeding deterrent effects of the plant materials.

The plant powders contain chemical compounds that may constitute excellent repellents and insecticides and can be employed in various storage environments. The *A. salicifolia* leaf powder and wood ash significantly repelled *S. zeamais* and *A. obtectus*, and the increase in repellent effect followed that of the concentrations. That repellent capacity varied with the types of powders. This repellence on *S. zeamais* was not influenced by the dosage, since no significant difference was observed among the different dosages of powders. All the dosages used significantly repelled the two insect pests. For *A. obtectus*, the repellence rate increased with increasing concentration of the leaf powder, while for wood ash, repellence was not dose‐dependent. This repellent ability of these powders is conferred by their chemical composition. The repellent property of the two plant powders improves their protective effectiveness by keeping grains out of the reach of insect pests and reducing their damage to the grains.

As emphasized by Nta et al. [40], the ability to reduce progeny production should be among the most important properties of an effective grain protectant. *A. salicifolia* leaf powder and wood ash significantly induced inhibition of F_1_ progeny production of *S. zeamais* and *A. obtectus*. This inhibition was dose‐dependent and varied according to the plant powders. Several studies revealed the inhibitory effect of plant powders on stored pest beetles in particular coleopteran pest insects of stored cereals and pulses. Goudoungou et al. [30] showed that *Chenopodium ambrosioides* and *Tephrosia vogelii* leaf powder inhibited F_1_ progeny production and adult emergence of the *A. obtectus* adults and Goudoungou et al. [29] showed that *Plectranthus glandulosus* leaf powder and *Hymenocardia acida* wood ash reduced F_1_ progeny production and then suppressed adult emergence of the *S. zeamais* adults. Leaf powder and wood ash of several plants have shown their ability to reduce the production of *A. obtectus* and *S. zeamais* F_1_ progeny. As presumed by Ntonifor et al. [41], *C. ambrosioides* leaf powder has negative effects on reproduction and development of immature stages in addition to its adulticidal activities. According to its phytochemical composition, due to several compounds that may have synergistic and/or potentiating interactions, leaf powder and wood ash are noxious both to adult and juvenile stages of insect pests. The mechanism can also be responsible for inhibiting the *A. obtectus* production of F_1_ as observed with the same plant concerning *S. zeamais*. The same assumption can be also made for *A. salicifolia*, even though these two plants do not belong to the same family. The tendency concerning adult mortality induced by the two plant powders was maintained for progeny inhibition; *A. salicifolia* wood ash was slightly more effective than the leaf powder for *A. obtectus*, while the two powders were less effective on *S. zeamais* with the wood ash being more effective than the leaf powder. The high mortalities of *A. obtectus* induced by *A. salicifolia* leaf powder and wood ash just 3 days after exposure significantly reduced the number of insects able to lay egg. By significantly reducing the number of eggs laid by the beetles, these substances inevitably induced the inhibition of progeny production. At 8 weeks after treatment, the leaf powder and wood ash tested in the present study were very effective in inhibiting the reproduction capacity and progeny emergence of *A. obtectus* with 100% inhibition at the highest doses, while for *S. zeamais,* wood ash was more active in impairing the reproduction of the insect pest than the leaf powder.

Plant‐based products can include botanical extracts, essential oils, plant powders and other natural compounds with insecticidal properties. These substances often contain bioactive compounds that repel or kill stored product insect pests [42]. The major constraints to the utilization of plant products in the field for stored products involve the low persistence, modifying contents of active ingredients, their difficult homologation as plant protectant, possible toxicity to humans and the effect on food taste [43]. The mechanisms by which plant‐based products control pests may involve repellence, insect growth regulation, interference with feeding or mating and direct toxicity. Understanding these mechanisms is crucial for optimizing their use. Plant‐based products may degrade more quickly than synthetic chemicals, requiring frequent reapplication [42, 43]. The effectiveness of plant‐based products may vary by region due to differences in climate, pest species and agricultural practices. Understanding these regional variations is important for widespread adoption. They may still leave residues on grains; it is essential to assess the safety of botanical residues and ensure they comply with regulatory standards [44]. Regular monitoring and evaluation of the effectiveness of plant‐based products are necessary to make adjustments to pest management strategies and improve outcomes over time. Then, it is interesting to combine plant products or extracts with other pest management techniques or strategies, such as sanitation, hermetic storage and trapping, in order to improve overall efficacy.

## 5. Conclusion

The leaf powder and wood ash of *A. salicifolia* have proven their insecticidal properties against *S. zeamais* and *A. obtectus* during storage by reducing grain weight loss and damage. These powders were toxic to maize weevils and bean beetles with the wood ash being more effective than the leaf powder. The plant powders were also effective at inhibiting progeny production, repelling the insects and preserving seeds for germination, with some discrepancies in the performance of the two powders (leaf powder and wood ash), as wood ash was more effective than the leaf powder. Thus, in storage structures of developing countries like Cameroon, wood ash could be considered as an effective alternative product in the preservation of the maize and beans because of their safety for consumers. This plant product might supersede the use of synthetic chemical insecticides. Additionally, to improve the usefulness of these findings, some investigations need to be carried out, especially regarding the persistence of the different products of A. salicifolia and their effectiveness against insect pests of other stored grains.

## Conflicts of Interest

The authors declare no conflicts of interest.

## Funding

This research work did not receive any specific grant from funding agencies in the public, commercial or not‐for‐profit sectors.

## Data Availability

The data sets used and/or analysed during this study are available from the corresponding author upon reasonable request.
